# On detection of periodicity in C-reactive protein (CRP) levels

**DOI:** 10.1038/s41598-018-30469-8

**Published:** 2018-08-10

**Authors:** Mohsen Dorraki, Anahita Fouladzadeh, Stephen J. Salamon, Andrew Allison, Brendon J. Coventry, Derek Abbott

**Affiliations:** 1 School of Electrical & Electronic Engineering, University of Adelaide, Adelaide, South Australia 5005 Australia; 2 Centre for Biomedical Electrical Engineering (CBME), University of Adelaide, Adelaide, South Australia 5005 Australia; 3 Department of Surgery & Tumour Immunotherapy Laboratory, University of Adelaide, Royal Adelaide Hospital, Adelaide, South Australia 5005 Australia

## Abstract

C-reactive protein (CRP) is an acute-phase plasma protein that can be used as a biomarker for activation of the immune system. A spectral analysis of CRP level over time for patients with gynaecological tumours has been reported by Madondo *et al*., using a periodogram method, suggesting that there is no significant periodicity in the data. In our study, we investigate the impact of low sample number on periodogram analysis, for non-uniform sampling intervals—we conclude that data of Madondo *et al*. cannot rule out periodic behaviour. The search for patterns (periodic or otherwise) in the CRP time-series is of interest for providing a cue for the optimal times at which cancer therapies are best administered. In this paper we show (i) there is no evidence to rule out periodicity in CRP levels, and (ii) we provide a prescription for the minimum data sample rate required in future experiments for improved testing of a periodic CRP signal hypothesis. The analysis we provide may be used for establishing periodicity in any short time-series signal that is observed without *a priori* information.

## Introduction

It has been proposed that the immune system exhibits fluctuation in its activity over time and that this can be characterised by oscillation of bio-markers that can measure inflammation, such as acute-phase serum markers and possibly body temperature^1,2^.

C-reactive protein (CRP), a widely used acute-phase protein, is a relatively sensitive objective serum bio-marker for systemic inflammation arising from immune system stimulation^3^. Inflammation can arise from conditions that stimulate the immune system such as infection and tissue damage, and has also been associated with advanced cancer in humans^1^.

There is a substantial body of literature describing the association between prognosis and C-reactive protein in patients with cancer. Note that CRP is widely reported in oncology as a marker for survival^4–14^, an indicator of cancer risk^15–21^, as a biomarker for tumour recurrence^22–31^, and as a reliable tool for making critical decisions in treatment^1,32,33^.

In a melanoma study, Coventry *et al*. suggested that CRP levels oscillate about a mean with a periodicity of approximately six-to-seven days^1^. The authors hypothesised that the CRP oscillations are part of a homeostatic immune response to advanced malignancy from preliminary data linking the timing of therapy to treatment success^1^.

Complete response (CR), where no cancer is detectable after therapy, is reported to be low after chemotherapy for most cancer types, with CR rates being of the order of 0–10%^34^. Establishing the character of the fluctuations associated with CRP might reveal a regular periodicity, which could provide an opportunity for exploring the efficacy of cancer-based therapies as a function of their timing relative to the patient’s own immune response oscillatory pattern^1^.

In another study, chemotherapy was initiated at the estimated peak of a CRP cycle and it was estimated that all patients exhibited oscillating CRP levels with an average periodicity of 7.8 days^35^. They conducted a pilot clinical study of twelve patients with metastatic melanoma who underwent serial CRP measurements every 2–3 days for two weeks. Both studies^1,35^ highlighted that the timing of either a vaccine-based therapy or cytotoxic chemotherapy relative to specific phases in the immune response cycle of a patient may possibly exert an impact on clinical outcome.

In order to establish the periodicity of CRP data, Madondo *et al*.^36^ employed a frequency domain analysis on a small number of CRP samples obtained at seven time-points over an interval of twelve days from a cohort of patients with gynaecological cancers. From individual patient serum CRP level data, periodograms were calculated and normalized to have the sum of squares equal to one, then averaged pointwise. The study was carried out under the null hypothesis that there was no consistent period to the CRP concentration, which implies no peaks in the mean periodogram beyond noise.

In contrast to the previous studies^1,35^, Madondo *et al*.^36^ concluded that CRP might not be a convenient bio-marker for the timing of cancer therapies, as their CRP data did not appear to oscillate periodically.

As will be shown, we find Madondo’s data does not contain enough number of data points to conclude whether the CRP data is periodic or not particularly for a hypothesised period of 7 days^1^. In our study, we perform a detailed statistical analysis because data is sampled in non-uniform time intervals in a signal where we have no *a priori* knowledge of periodicity, rather than utilising a simplistic application of Nyquist’s sampling theorem^37^ that does not apply to this case.

The rest of paper is organized as follows. In the next section we outline our statistical approach. Next, we study the impact of undersampling of a known sine wave on its corresponding periodogram. Moreover, we demonstrate use of the Scargle’s significance test for examining the significance of the period obtained from the peak of a periodogram.

## Periodogram analysis of CRP

There are numerous mathematical methods for extracting the periodicity of a biological signal; however, dealing with an unevenly sampled biological signal that is contaminated with noise is challenging. Moreover, when there is no *a priori* information on whether the signal is periodic or not, this adds to the difficulty.

The periodogram is a convenient method for estimating spectral density of a signal, and it is widely used for detecting spectral information in signals with noisy conditions. However, studies show that the periodogram has limitations in handling non-sinusoidal waveforms and sparse signals^38^.

A periodogram analysis on the variation of CRP over time for patients with gynaecological tumours has been reported, suggesting that there is no periodicity in CRP data^36^. In that study, by Madondo *et al*., CRP samples were collected from a cohort of nineteen patients, logging only seven CRP data points over an interval of twelve days. We suggest that seven samples is too small a sample quantity to capture the hypothesized periodicity^1^ over twelve days.

A sinusoid models an ideal periodic signal and this is often seen in the bio-signal processing literature^39,40^. In order to demonstrate our assertion that the CRP time-series of Madondo *et al*. is undersampled, we first examine a known ideal sine wave. We randomly sample the sine wave with two randomly placed samples in a period and then estimate the period using a periodgram–we then repeat this using three random samples and so on up to 40 random samples.

Noisy and under-sampled data produce noisy periodograms with spurious peaks. Therefore, the peaks may not be due to the presence of any actual periodic component. Spurious peaks can be surprisingly large, thus it is vital to apply a reliable test in order to detect actual periodicity. Periodogram’s peaks significance assessment methods such as Fisher *g*-test^41,42^ and Scargle’s significance^40^ tests have been used to investigate periodic components in time-series. The general approach of Fisher’s *g*-test together with multiple testing correction for the detection of periodic time-series is followed in a gene expression study^43^. Moreover, the combination of a Scargle test statistic and a multiple hypothesis testing procedure is suggested to detect significant periodic gene expression patterns^40^. The tests determine the significance of the estimated period that allows one to distinguish periodic from purely random processes. In this paper, we use Scargle’s significance test in each case to assess the periodogram peaks.

### Periodogram of unevenly sampled sine waves

Different types of periodograms are introduced by previous studies for examining frequency-domain models of time-series. In the paper by Madondo *et al*.^36^ the type of periodogram used is unspecified, however, the Lomb-Scargle periodogram^44^ is applied in our work. One limitation of a basic periodogram is that it requires uniformly sampled data^45^. The data produced by the patient sampling in Madondo’s study are unevenly spaced in time. This is addressed by use of the Lomb-Scargle periodogram that is a convenient approach for finding periodicity in non-uniformly sampled time-series.

**Figure 1:**
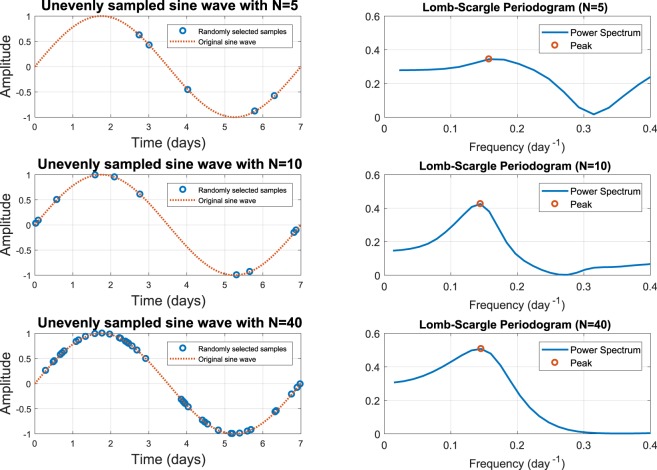
Unevenly sampled sine waves, with a single period of seven days, are shown on the left. The corresponding periodograms are plotted on the right. Peaks with a period of seven days (ie. equal to a frequency of about 0.14 days^−1^) can be seen in the periodograms. For low sample numbers, these peaks are very broad and are difficult to resolve. When the sample rates are higher, the peaks are much more distinct, and easy to detect.

In order to investigate the impact of periodogram undersampling, Fig. 1 shows the periodogram of a sine wave with *N* = 5, 10 and 40 samples respectively, in a period. For a sample size of *N* = 5, the peaks in the periodogram are not significant. It may be seen that with larger sample sizes the peaks in the periodogram become more pronounced.

**Figure 2:**
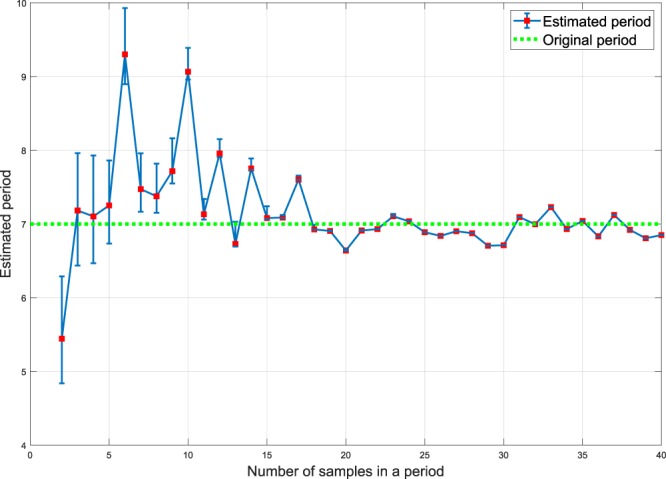
Estimated period using the Lomb-Scargle periodogram for a randomly sampled sine wave versus different numbers of samples in a period. The random sampling procedure is carried out twenty times for each sample number–the averages of the twenty results are plotted as red squares, and the error bars are calculated based the actual range of values observed. Clearly, periodogram estimation of period fails for low sample numbers.

To examine the impact of sampling number, the periodogram method is applied to ideal sine waves with different numbers of samples in a period (two to 40) and period of seven days. The result is shown in Fig. 2. The random sampling procedure is carried out twenty times for each individual number of samples. Considering Fig. 2, an increase in the number of samples in a period results in reduced error of the estimated period. In fact, the figure reveals the periodogram is sensitive to unevenly sampled data with low numbers of samples in a period. This indicates that the CRP data^36^ suffers from an insufficient sampling rate. In other words, the CRP data in^36^ does not have enough number of samples to conclude whether the CRP time-series is periodic or not.

### Significance test

Spurious peaks arise in periodograms of the data, not because of any periodic component in the data, but because of the noise components. Thus it is essential to apply a reliable test, to determine whether the peaks are significant. In this section, the relevance of periodogram peaks and impact of number of samples on the peaks is assessed, and in particular the Scargle’s significance test is used to test significance.

The null distribution of the Lomb-Scargle periodogram *Z*_*j*_ = *P*(*ω*_*j*_) at a given frequency *ω*_*j*_ is exponentially distributed^46^, i.e. the cumulative distribution function (CDF) of *Z*_*j*_ is

1 $$\begin{array}{rcl}F(z) & = & {\rm{\Pr }}[{Z}_{j}\le z]\\  & = & 1-{e}^{-z},\end{array}$$

for *j* = 1, 2, 3, …, *M* (the number of test frequencies). The null hypothesis is that there is no consistent period in the data, versus the alternative that the data is periodic.

In the case, that the peak in the periodogram is attained at frequency *ω*_*k*_ among *M* independent frequencies, we denote such a peak by *X* = max_*j*_*P*(*ω*_*j*_) = *P*(*ω*_*k*_). Then, for independently normally distributed noise, if there were *M* independent frequencies to test, the probability that the peak periodogram *X* is smaller than *x* is obtained by

2 $$\begin{array}{l}{\rm{\Pr }}[X\le x]={\rm{\Pr }}[{Z}_{j}\le x,\,j=1,2,3,\mathrm{...},M]={\mathrm{(1}-{e}^{-x})}^{M}.\end{array}$$

Therefore, the statistical significance level, the *p*-value, for testing the null hypothesis that such a peak in periodogram is due to chance is given by

3 $$\begin{array}{l}p \mbox{-} \mathrm{value}=1-{(1-{e}^{-x})}^{M}.\end{array}$$

The function is proposed as a false alarm probability function by Scargle^46^. Therefore, for each individual periodogram, Equation 3 generates a *p*-value that allows us to test whether it exhibits any periodicity, i.e. whether the maximum peak in the periodogram is significant.

In summary, the time-series methodology proposed here consists of the following steps:

- Apply the periodogram method to check graphically whether the data are periodic or not.
- Apply the Scargle’s significance test in each case, and calculate the corresponding *p*-value for each test.
- The *p*-values under 0.05 reject the null hypothesis that the data are random, i.e. the obtained periodicity is significant.

**Figure 3:**
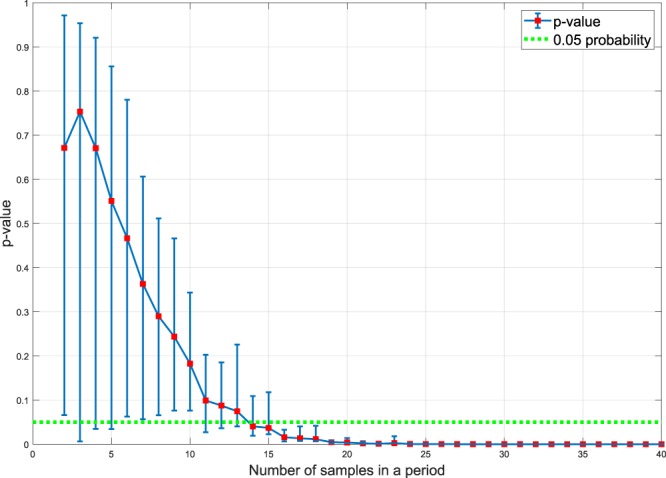
The corresponding *p*-values for a randomly sampled sine wave as a function of the numbers of samples in a period. The sampling procedure is carried out twenty times for each individual number of samples. Only the points under the green line indicate statistically significant periodicity. The error bars are calculated based on the actual range of values observed.

Figure 3 demonstrates corresponding *p*-values for the periodogram of an ideal sine wave with different numbers of samples in a period (two to 40) that is shown in Fig. 2. In this case the level of significance is set at *p* = 0.05.

The points under the green dotted line in Fig. 3 reject the null hypothesis. It can be seen that an increase in the numbers of samples in a period results in reduced *p*-value to zero. In fact, the significance test demonstrates that the periodogram is dramatically sensitive to the unevenly sampled signal with low numbers of samples in a period. This shows that in the periodogram study of Madondo *et al*.^36^ using the methodology described, the resulting data suffers from an insufficient sampling rate when attempting to detect periods.

The following question can be posed: Can the sampling pattern in the Madondo *et al*. study detect hidden periodicity more or less than seven days? To find the answer, we investigate sine waves with varying periods from 2 to 15 days with the sampling pattern used in Madondo’s study, and carry out the periodogram analysis and the Scargle significant test.

**Figure 4:**
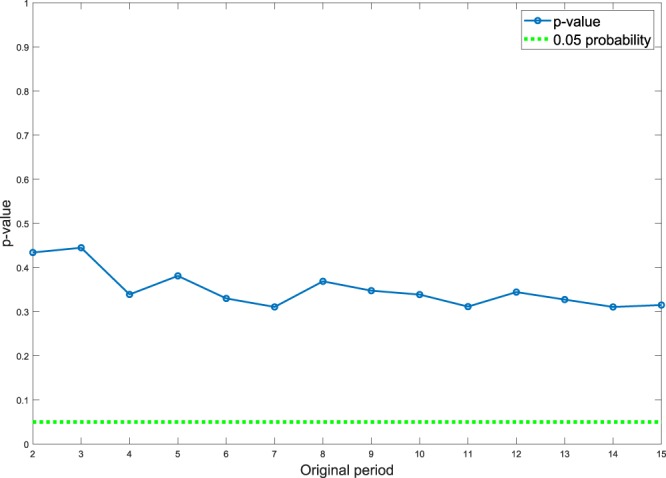
The corresponding *p*-values for sine waves with different periods from 2 to 15 days and with the sampling pattern used in Madondo’s study is demonstrated. All the points are above of the green dotted line that means Madondo’s approach is not able to detect significant periodicity in this range.

The corresponding *p*-value for the sine waves with different periods from 2 to 15 days is shown in Fig. 4. To simulate Madondo’s data set, the samples are selected on days 1, 3, 5, 6, 8, 10 and 12. From Fig. 4 it is obvious that the *p*-values for all the periods are not able to reach the significance level (the green dotted line).

### Mean periodogram

Before applying a significance test, Madondo *et al*.^36^ averaged the periodograms. The mean periodogram is a simple extension of the standard periodogram, and it is widely used in time-series analysis^41^. However, the mean periodogram is a method for dealing with a large number of signals, yielding accurate results only when a number of the time-series exhibit identical periodic behaviour. Moreover, it has been demonstrated that the mean periodogram is sensitive to sample size, and the tool cannot estimate the dominant periodicity with a low number of samples^41^.

To examine the impact of variation in periodic behaviour, the mean periodogram is applied to two sets of test data consisting of 19 time-series with (i) random samples and with (ii) purely sinusoidal values. The particular value, of 19 samples, is chosen to simulate Madondo’s data set containing 19 patients. The random series possess a normal distribution, and the sinusoidal series possess varying periods with an average of seven days and standard deviation of three days. In accordance with Madondo’s study, individual periodograms are calculated and standardised to have a sum of squares equal to 1 then averaged pointwise.

**Figure 5:**
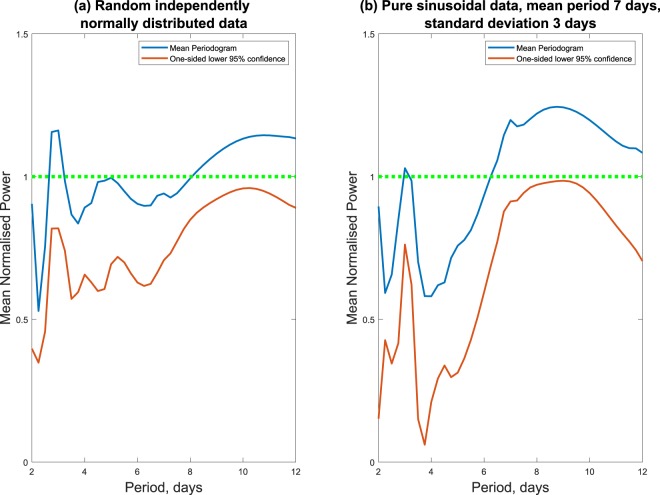
The periodicity for (**a**) 19 purely random time-series and (**b**) 19 purely sinusoidal time-series are assessed using the null hypothesis that there is no periodicity. The pointwise lower one-sided 95% confidence bound (red line) needs to exceed the null line (green dotted line) to suggest a significant peak. What this figure illustrates is that Madondo’s mean periodogram approach may not be able to indicate periodicity in time-series with varying periods.

The lower one-sided 95% confidence bound for the mean periodogram is calculated. Figure 5 shows that the one sided lower 95% confidence indicated with red line is under the green dotted line for both random series and ideal sine waves. The null hypothesis that there is no periodicity, is not rejected if the red line is below the green dotted line in Fig. 5. Thus, mean periodogram analysis with the sampling pattern used in Madondo’s study may not be able to determine periodicity from 19 pure sine waves with varying periods.

## Conclusion

Although CRP has been used as a biomarker of infection, inflammation and tissue damage, multiple sequential measurements of CRP close together in time are essential for the understanding of its behaviour as a function of time. It seems likely that immunotherapy and chemotherapy treatments can be optimized, by correctly estimating the times of the treatments. It will assist optimization of these times if cyclic behavior is correctly and accurately identified, and those cycles cannot be correctly identified without a sufficient number of samples within a defined measurement period.

In our simulation studies we investigate the periodogram-based CRP study of Madondo *et al*.^36^, and we show that the periodogram approach of Madondo is sensitive to sparse sampling and that there are an insufficient number of samples for establishing the existence of periods using their methodology as described.

Moreover, the number of samples in Quevedo’s study^35^ is insufficient to confirm 7-day periodicity. Thus, the 7-day hypothesis has neither been statistically confirmed nor ruled out.

In our study, the Lomb-Scargle periodogram is applied to a non-uniformly sampled ideal sine wave. Application of the Scargle’s significance test and the calculation of *p*-values clearly shows that the accuracy of the periodogram analysis is dramatically sensitive to low sample size.

The significance of establishing the presence of CRP cycles is that it opens up avenues for exploring the efficacy of cancer-based therapies as a function of their timing relative to the biological fluctuations in the immune response occurring in the individual cancer patient. Furthermore, CRP measurement is a low-cost and rapid approach and can be performed conveniently, potentially leading to significantly improved outcomes related to the timing of cancer therapies.

Finally, our results provide a prescription for future CRP studies showing how many samples are required to establish any hypothesized periodicity. For 95% confidence, as many as 14 samples per period are required. For the case of CRP, such a large number of samples is presently difficult to carry out at the clinical level principally for practical reasons. However, this may become possible in the future when improvements in technology can provide continuous point-of-care CRP measurement, or with alternative means of continuous or near continuous *in vivo* techniques of measurement of immune system activity in a clinical setting.

## Electronic supplementary material

LaTeX Supplementary File

LaTeX Supplementary File

LaTeX Supplementary File
